# Comparison of Clinical Characteristics of Patients with Asymptomatic vs Symptomatic Coronavirus Disease 2019 in Wuhan, China

**DOI:** 10.1001/jamanetworkopen.2020.10182

**Published:** 2020-05-27

**Authors:** Rongrong Yang, Xien Gui, Yong Xiong

**Affiliations:** 1 Department of Infectious Diseases, Zhongnan Hospital, Wuhan University, Wuhan, China

## Abstract

This case series examines clinical characteristics of patients with asymptomatic vs symptomatic coronavirus disease 2019 in Wuhan, China.

## Introduction

Coronavirus disease 2019 (COVID-19) emerged in Wuhan, China, in December 2019 and has spread globally with sustained human-to-human transmission outside China.^1,2^ To control the spread of COVID-19 and isolate patients as early as possible, the Chinese government requested that close contacts of individuals with COVID-19 must be screened for severe acute respiratory syndrome coronavirus 2 (SARS-CoV-2) infection. During the screening process, we found some patients whose test results were positive for SARS-CoV-2 but who had no symptoms or signs throughout the course of the disease. Considering that little is known about the differences of clinical features and prognosis between patients who were asymptomatic vs those who were symptomatic,^3,4^ this case series aimed to describe the clinical characteristics of patients with SARS-CoV-2 infection confirmed by reverse transcription–polymerase chain reaction (RT-PCR) from 26 transmission cluster series in Wuhan, China, from December 24, 2019, to February 24, 2020.

## Methods

This case series was approved by the institutional ethics board of Zhongnan Hospital of Wuhan University. All consecutive patients with COVID-19 confirmed via RT-PCR admitted to Zhongnan Hospital of Wuhan University from December 24, 2019, to February 24, 2020, were enrolled. Oral informed consent was obtained from all patients. Epidemiological, symptoms, signs, laboratory values, chest computed tomography (CT) scans, treatment measures, and outcomes data during the hospital stay were collected. Nasopharyngeal swab samples were collected for extracting SARS-CoV-2 RNA from patients suspected of having SARS-CoV-2 infection.

The indicative patients were recruited from 26 cluster cases and had confirmed history of exposure to the Hunan seafood market or had close contact with another patient who had been hospitalized for COVID-19, and they were confirmed to have SARS-CoV-2 infection by RT-PCR from nasopharyngeal swabs. Routine chest CT scans and SARS-CoV-2 testing from nasopharyngeal swab were performed on their close contacts. The close contacts with other exposure histories were excluded. The included patients who were exposed to the same indicative patients with COVID-19 were defined as clustered cases, and the date of initial exposure was identified to determine incubation period.

Data were analyzed using SPSS statistical software version 19.0 (IBM). Categorical variables were described as frequency rates and percentages, and continuous variables were described using mean, median, and interquartile range (IQR) values. χ^2^ analysis was conducted to examine the categorical variables. Means for continuous variables were compared using independent group *t* tests when the data were normally distributed; otherwise, the Mann-Whitney *U* test was used. All *P* values were 2-tailed, and *P* < .05 was considered to be significant.

## Results

This case series includes data for 78 patients from 26 cluster cases of exposure to the Hunan seafood market or close contact with other patients with COVID-19. All patients were confirmed to have SARS-CoV-2 infection by RT-PCR from nasopharyngeal swabs. The median (IQR) number of patients per cluster was 3 (2-3) patients, and the range was 2 to 10 patients per cluster.

The 78 close contacts confirmed with SARS-CoV-2 infection were hospitalized in same medical area and provided the same treatments administered by the same health care workers. A total of 33 patients (42.3%) were asymptomatic, while 45 patients (57.7%) were symptomatic. The symptoms and signs, such as fever, fatigue, and dry cough, were monitored every day. Detecting SARS-CoV-2 from nasopharyngeal swab was monitored every 24 to 48 hours. For patients with stable conditions, a second chest CT was conducted 4 to 6 days after the first time, then 6 to 7 days after the second time. Chest CT was also conducted at any time a patient’s condition became worse. CD4^+^T lymphocyte count was tested every 5 to 6 days.

Data about clinical characteristics of patients with asymptomatic and symptomatic SARS-CoV-2 infection are presented in the Table. Patients who were asymptomatic, compared with patients with symptomatic SARS-CoV-2 infection, were younger (median [IQR] age, 37 [26-45] years vs 56 [34-63] years; *P* < .001), and had a higher proportion of women (22 [66.7%] women vs 14 [31.%] women; *P* = .002), lower proportion of liver injuries (1 patients [3.0%] vs 9 patients [20.0%]; *P* = .03), less consumption of CD4^+^T lymphocytes (median [IQR] CD4 lymphocyte count during recovery, 719 [538-963] per μL vs 474 [354-811] per μL [to convert to to ×10^9^ per liter, multiply by 0.001]; P = .009), faster lung recovery in CT scans (median [IQR] duration, 9 [6-18] days vs 15 [11-18] days; *P* = .001), shorter duration of viral shedding from nasopharynx swabs (median [IQR] duration, 8 [3-12] days vs 19 [16-24] days; *P* = .001), and more stable results of SARS-CoV-2 testing (4 fluctuated results [12.1%] vs 15 fluctuated results [33.3%]).

**Table.** Clinical Features and Prognosis of Patients With Coronavirus Disease 2019 From 26 Transmission Cluster Series

Characteristic	Patients, No. (%)		χ^2^ or *F* test	*P* value
	Asymptomatic (n = 33)	Symptomatic (n = 45)		
Age, median (IQR), y	37 (26-45)	56 (34-63)	10.221	.001
Women	22 (66.7)	14 (31.1)	9.685	.002
Incubation period, median (IQR), d	NA	3 (2-6)	NA	NA
Baseline liver injury^a^	1 (3.0)	9 (20.0)	4.905	.03
Duration of viral shedding, median (IQR), d^b^	8 (3-12)	19 (16-24)	7.022	.001
Duration of lung recovery, median (IQR), d^c^	9 (6-18)	15 (11-18)	6.914	.001
Maximum difference of CD4 lymphocytes during treatment, median (IQR), /μL^d^	203 (170-304)	328 (145-506)	4.570	.04
CD4 lymphocyte count during recovery, median (IQR), /μL^e^	719 (538-963)	474 (354-811)	7.203	.009
Fluctuated results of SARS-CoV-2 test^b^	4 (12.1)	15 (33.3)	4.649	.03
Deaths	0	2 (4.4)	1.505	.22

Abbreviations; IQR, interquartile range; NA, not applicable; SARS-CoV-2, severe acute respiratory syndrome coronavirus 2.

SI conversion factor: To convert lymphocytes to ×10^9^ per liter, multiply by 0.001.

^a^ Liver injury was defined as serum alanine aminotransferase/aspartate aminotransferase levels more than 1.5 U/L (to convert to microkatals per liter, multiply by 0.0167).

^b^ Measured via nasopharynx swab.

^c^ Defined as when lung lesions began to be absorbed or contracted, observed by chest computed tomography.

^d^ Treatment period was defined as the time from admission to the occurrence of 2 consecutive negative results for SARS-CoV-2 from nasopharyngeal swab.

^e^ Recovery period was defined as the time from the end of treatment period to when chest computed tomography results were within reference ranges.

## Discussion

Our finding of less consumption of CD4^+^T lymphocyte in asymptomatic infections suggests that damage to the immune system in asymptomatic infections was milder compared with symptomatic infections. Although patients who were asymptomatic experienced less harm to themselves, they may have been unaware of their disease and therefore not isolated themselves or sought treatment, or they may have been overlooked by health care workers and thus unknowingly transmitted the virus to others. Fortunately, patients with asymptomatic SARS-CoV-2 infection have a shorter duration of viral shedding from nasopharyngeal swabs and lower risk of a recurring positive test result of SARS-CoV-2 from nasopharyngeal swabs, which can provide a reference for improving the prevention and control strategies for patients who are asymptomatic.

This study has some limitations. Although all patients whose RT-PCR tests were positive for SARS-CoV-2 had been exposed to same patients, were hospitalized in same medical area, and provided the same treatments administered by the same health care workers, the clinical differences between patients who were asymptomatic vs those who were symptomatic could have been more objectively observed.

Since patients with asymptomatic COVID-19 were relatively concealed, the fact of viral shedding detected via nasopharyngeal swabs must not be ignored. Therefore, identifying and isolating patients with asymptomatic COVID-19 as early as possible is critical to control the transmission of COVID-19. Close contacts of patients with COVID-19 should be closely monitored to avoid secondary transmission.
