# Incorrect Reporting of Statistical Tests

**DOI:** 10.1001/jamanetworkopen.2020.14310

**Published:** 2020-06-26

The Research Letter, “Comparison of Clinical Characteristics of Patients with Asymptomatic vs Symptomatic Coronavirus Disease 2019 in Wuhan, China,”^1^ published on May 27, 2020, incorrectly reported statistical tests in the Methods section and findings in the Table. The correct tests are as follows: “χ^2^ analysis was conducted to compare the distributions of categorical variables (ie, sex, baseline liver injury, fluctuated results of SARS-CoV-2 test, and death) between asymptomatic and symptomatic patients. Continuous variables (ie, age, duration of viral shedding, duration of lung recovery, maximum difference of CD4 lymphocytes during treatment, and CD4 lymphocyte count during recovery) were compared using *t* tests.” The findings have been corrected in the Table and are similar to the originally reported comparisons, and the statistical results continue to indicate *P* < .01. This article has been corrected.
